# Adolescents with full or subthreshold anorexia nervosa in a naturalistic sample: treatment interventions and patient satisfaction

**DOI:** 10.1186/s13034-020-00323-9

**Published:** 2020-05-02

**Authors:** Katarina Lindstedt, Emma Forss, Marie Elwin, Lars Kjellin, Sanna Aila Gustafsson

**Affiliations:** 1 University Health Care Research Center, Faculty of Medicine and Health, Örebro University, Örebro, Sweden; 2 Faculty of Medicine and Health, Örebro University, Örebro, Sweden

**Keywords:** Adolescents, Anorexia nervosa, Naturalistic sample, Treatment, Patients’ perspectives, Cluster analysis

## Abstract

**Background:**

Despite major research efforts, current recommendations of treatment interventions for adolescents with anorexia nervosa are scarce, and the importance of patient satisfaction for treatment outcome is yet to be established. The overall aim of the present study was to examine treatment interventions and patient satisfaction in a naturalistic sample of adolescents with anorexia nervosa or subthreshold anorexia nervosa and possible associations to outcome defined as being in remission or not at treatment follow-up.

**Methods:**

Participants were identified through the Swedish national quality register for eating disorder treatment (SwEat). The samples consisted of 1899 patients who were follow-up registered 1 year after entering treatment and 474 patients who had completed a 1-year patient satisfaction questionnaire. A two-step cluster analysis was used for identifying subgroups of patients who received certain combinations and various amounts of treatment forms.

**Results:**

Patients who received mainly family-based treatment and/or inpatient care were most likely to achieve remission at 1-year follow-up, compared to patients in the other clusters. They were also younger, in general. Individual therapy was the most common treatment form, and was most appreciated among the adolescents. At 1-year follow-up, many patients reported improvements in eating habits, but far fewer reported improvements regarding cognitive symptoms. Overall, the patients rated the therapist relationship in a rather positive way, but they gave quite low ratings to statements associated with their own participation in treatment.

**Conclusions:**

The results indicate that young adolescents who receive mainly family-based treatment and/or inpatient care respond more rapidly to treatment compared to older adolescents who receive mainly individual therapy or mixed treatment interventions. At 1-year follow-up, the adolescents reported improvements in behavioral symptoms and seemed quite satisfied with the therapist relationship.

## Background

Anorexia nervosa (AN) most often has its onset in adolescence [1] and might lead to severe consequences for many young women and men [2, 3]. AN affects mainly girls and young women, and lifetime prevalence is about 3–6 times higher for women than men [4, 5]. However, the rates among boys and young men are increasing [6, 7]. The incidence is the highest between the ages of 15–19, for both genders [8]. Comorbidity between AN and other psychiatric disorders, e.g. anxiety disorders and mood disorders, is common [6, 9].

Most treatment for eating disorders (ED) is interdisciplinary and offered at specialized ED units, where different professions work together in teams [10–12], although there are only a few uniform recommendations on what interventions to use for different patients in different situations [10, 13]. According to Swedish clinical guidelines, patients are usually offered some form of “main treatment,” either individual, together with family, in a group with other patients or mixed [11]. The main treatment is often supplemented by different kinds of additional interventions. Most individuals with AN can be treated in outpatient care, but brief inpatient care might be necessary for patients with a severe illness, e.g. [10, 11].

Individual therapy is a treatment form where the patient has individual meetings with a therapist, often including counseling and psychoeducation [10, 14, 15]. Common forms of individual treatment are interpersonal psychotherapy [16], adolescent-focused psychotherapy or enhanced cognitive behavioural therapy [10]. Among adolescents, family-based treatment has the strongest empirical support for patients with a restrictive symptomatology [5, 10, 15, 17, 18]. Family-based treatment implies meetings with a therapist in which also the patient’s parent/s, and sometimes siblings, are involved. The treatment is often based on systemic, cognitive behavioral or psychodynamic principles [19]. Apart from a general focus on ED symptoms, family-based treatment have a strong focus on how the illness affects family relationships and how the parents can support their child’s recovery [10, 11, 15]. Group therapy, where one or two therapists meet a group of patients together, is often based on a shared symptomatology and offered to patients who might benefit from exchanging experiences with others in group sessions [11]. Patients with a restrictive symptomatology are not the main target group for such therapy [20], although multi-family therapy, based on conjoint sessions with three or four patients and their families, is a treatment form of increased interest for adolescents with restrictive ED [21, 22]. Complementary interventions include diet counseling (in which the patients meet with a dietician), meal training, physiotherapy, medical treatment and somatic treatment with a focus on physical health issues caused by the ED [11].

Although family-based treatment has proven to be the most effective intervention for adolescents with AN [10, 17, 18], it is not always appreciated by adolescents [23–26]. In general, young patients rate individual therapy higher than family-based treatment [23, 24, 26, 27] and put strong emphasis on the therapist relationship [26, 28–30]. Studies of treatment satisfaction among patients with AN are rather uncommon and have shown diverse results. Some studies on adolescents propose that they are generally quite unsatisfied with, or have mixed opinions about their treatment [23–25], while other studies indicate that the majority of patients are quite satisfied [27, 28]. Therapists’ genuine engagement in treatment and ability to focus on “the whole patient” have been shown to contribute to high patient satisfaction [30], while the opposite is the case for e.g. distrust and experienced lack of information in treatment [26]. Dissatisfaction with treatment has been associated with slow treatment progress and premature termination [25, 31] and might lead to affective symptoms such as anxiety, depression, and loneliness, which increases the risk for weight loss and relapse [32]. Patients’ own opinions, together with research evidence and clinical expertise, are useful when trying to improve existing interventions and developing new forms of treatment, according to the three-legged stool of evidence-based practice in ED treatment [31].

A previous study on adolescents registered in SwEat showed a relatively good outcome for adolescents with AN or subthreshold AN, and the results indicated that treatment for this group of patients has become more effective over the past 15 years [33]. However, it did not examine what treatment interventions the patients received or the patients’ own perspectives, which is the focus of the present study. Since many patients in clinical practice receive more than one type of treatment, our intention was to look at possible differences across patients with different treatment combinations.

The overall aim of the present study was to examine treatment interventions and patient satisfaction in a naturalistic sample of adolescents with AN or subthreshold AN and possible associations to outcome defined as being in remission or not at treatment follow-up. The study is based on the following questions:

- What are the most common treatment forms?
- Do different combinations of treatment forms and/or differences in health-care consumption predict remission at treatment follow-up?
- How do the adolescents evaluate their treatment after 1 year, and what aspects of treatment were most important for them?

## Methods

This is a naturalistic study based on data from the Swedish national quality register for eating disorder treatment (SwEat). SwEat is a longitudinal internet-based quality assurance register with the objectives to document clinically important key variables such as health-care consumption, treatment duration, different types of treatment interventions, and treatment outcome [34]. A total of 108 units, including all specialized ED units in Sweden and a fair number of general psychiatric units, participated in SwEat between 1999 and 2014 [35]. It has been suggested that studies with a naturalistic design can provide valuable knowledge about results of different treatments in real-life settings, as a complement to more commonly used randomized controlled studies [36, 37].

The objective of SwEat is to gather information about a treatment episode, from when a patient is entering treatment until completion of treatment, and patients are included regardless of age and gender. Information is initially registered in SwEat when a patient is entering treatment, provided that the unit intends to commence treatment, and the patient (or guardians, when the patient is underage) has given her/his consent to registration. A total of 17,611 initial registrations were made in SwEat between 1999 and 2014 [35]. If a patient terminates treatment and later on enters a new treatment episode, information in SwEat is registered again on the same occasions, which means that an individual might be initially registered in SwEat more than once. However, in the present study, solely information about the first treatment episode was included for patients who had more than one episode registered. After initial registration in SwEat, the patient is followed up once a year until end of treatment (EOT) [34]. During the years examined, at the time of follow-up, the patients also received a patient satisfaction questionnaire from the SwEat administration, to fill in and send back [35].

### Study sample

**Fig. 1:**
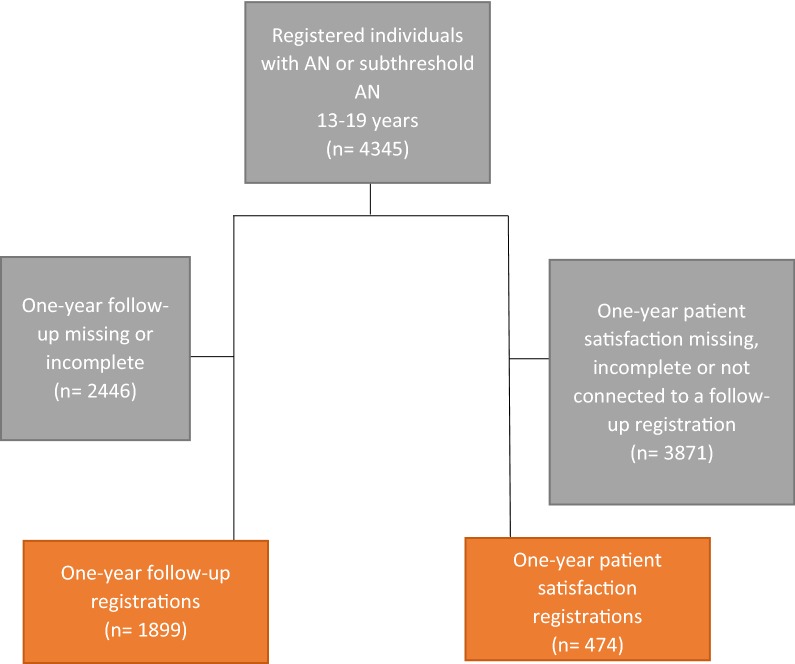
Flowchart, patients registered in SwEat during 1999–2014

A total of 4345 individuals in the age range 13–19 were initially registered at one of the participating treatment units between 1999 and 2014, and diagnosed with AN or subthreshold AN, according to Diagnostic and Statistical Manual of Mental Disorders IV (DSM-IV) [38] (see Fig. 1 for a flowchart). A total of 72 treatment units were represented in the study, 40 of which were specialized ED units. Of 4345 individuals, 1899 were follow-up registered 1 year after entering treatment, thus available for analyses in this study. When examining patient satisfaction, we included the 474 individuals who had completed a 1-year patient satisfaction registration. Most of the patients in both study populations were women (94.1% and 98.1%), diagnosed with AN (55.3% and 61.6%). The mean age at treatment onset was 16.1 and 16.3, and mean BMI was 17.2 and 17.1.

A more detailed description of the collected data in SwEat is to be found in a previous study [33]. According to that study, 35% of the initially registered patients in this age and diagnosis group were lost to follow-up in SwEat. Comparisons between the followed-up patients and the ones who were lost to follow up regarding baseline characteristics showed that followed-up patients had a later ED onset, a shorter illness duration, and had more social complications at treatment onset [33].

Experienced staff in multidisciplinary teams diagnosed the patients. As of 2008, at most Swedish ED units, diagnoses are made on the basis of the Structured Eating Disorder Interview (SEDI) [39]. Before that, a commonly used interview guide was the Rating of Anorexia and Bulimia interview (RAB) [40]. During the years examined, DSM-IV [38] constituted the basis for diagnoses at Swedish ED units.

Previous studies have shown that patients with subthreshold AN, defined as patients diagnosed with eating disorder not otherwise specified (EDNOS), not fully meeting the criteria for AN, often suffer from symptoms to the same extent as patients with AN [41–43]. Also, in clinical practice they are most often subject to the same interventions as patients with AN. Based on these results, and on the fact that the AN criteria are revised and broadened in Diagnostic and Statistical Manual of Mental Disorders 5 (DSM-5) [44], we included patients with subthreshold AN.

### Measures

Patients in the present study were examined based on follow-up registrations, monitored by therapists, and patient satisfaction questionnaires, in which the patients evaluated their treatment based on a number of statements. Both were conducted approximately 1 year after treatment onset. Data regarding baseline characteristics, outpatient treatment, inpatient care, health-care consumption, premature termination of treatment and patient satisfaction were explored. Outpatient treatment included individual therapy, family-based treatment, group therapy and complementary interventions. Inpatient care was received at either a specialized ED department, a general psychiatric department or a general somatic department. Health-care consumption was measured in number of outpatient sessions, number of days in inpatient care and number of days with medical treatment from initial registration. Premature termination of treatment was defined as discontinuation of treatment on patients’ or parents’ initiative. Information about patient satisfaction was collected through a questionnaire that was sent home to the patients from the SwEat administration, at the time of 1-year follow up. It included questions about the value of different treatment forms, patients’ perceptions of their therapist/s, perceived improvements in symptoms over the last year, and importance and fulfillment of treatment goals.

Register variables are presented in Additional file 1. In the analyses, a few of the original answer alternatives in the patient satisfaction questionnaire were combined into broader categories, which is also presented in Additional file 1.

In analyses of the follow-up registrations regarding treatment interventions the patients were categorized into three different age groups. These were; 1) 13–14 years (n = 434); 2) 15–17 years (n = 970) and 3) 18–19 years (n = 495).

The main outcome measure was being in remission or not at 1-year follow-up. Remission was defined as not fulfilling criteria for any ED diagnosis, which was assessed by experienced staff. Other variables selected as outcome measures were weight status-defined as low or normal weight, based on the BMI percentile method for calculating expected body weight [45] and adjusted for age and gender according to Swedish reference values [46], sick leave-defined as full or part-time sick-leave from work or school, and treatment completion-defined as completion of treatment in agreement between patient and therapist.

Most of the variables in the follow-up registrations have 1–3% missing or invalid answers. In the patient satisfaction questionnaire, which is sent home to the patients and answered manually, most of the variables have 1–9% missing or invalid answers. In a few cases the proportion is up to 30%, and regarding questions about important treatment goals, a mean of 19% of the answers were missing or invalid. In the analyses, the missing values were excluded.

### Statistical analyses

Statistical analyses were carried out using IBM SPSS Statistics 23 [47]. Treatment interventions were examined by using frequencies to determine the ratio of patients who received different interventions. In order to identify subgroups of patients who received certain combinations and various amounts of treatment forms, cluster analysis was used.

Cluster analysis is a method that can be used to identify groups of individuals in a sample who share a certain profile [48]. As the data in the present study included both binary and continuous variables, a two-step cluster analysis method was used, which is also suitable for large sets of data [48, 49]. The individuals were grouped together based on received treatment in the following forms: individual therapy, family-based treatment, group therapy, complementary interventions and inpatient care. The amount of health care consumption (basic interventions and complementary interventions) was also included in the analysis. The cluster solutions were compared using Schwarz’s Bayesian Criterion (BIC), and the log-likelihood was used as distance measure. Cluster data were compared through Pearson’s Chi square test, independent-samples *t* test and analysis of variance (ANOVA) tests as appropriate.

The clusters from the two-step cluster analysis were used in the next step as predictors in a logistic and multiple logistic regression analysis together with the following variables, possibly associated with treatment outcome [33]: age, diagnosis (AN or subthreshold AN), and weight status (low or normal weight) at treatment onset, and premature termination of treatment (on patients’ or parents’ initiative). This was done in order to observe if certain combinations and various amounts of treatment forms could predict remission at 1-year follow-up.

Statements in the patient satisfaction questionnaire were examined using descriptive measures to gather percentage and mean numbers. When examining differences between clusters regarding patient satisfaction of treatment interventions, we used paired-samples t-test. Variations between patients who terminated treatment prematurely and patients who followed treatment according to plan, were examined through Pearson’s Chi square test, independent-samples t-test, and ANOVA.

A nonparametric test, Related-Samples McNemar test, was used when examining differences between the proportions of patients that felt a certain goal was important and the proportions that considered it fulfilled.

Thresholds were set at *p* < 0.001 throughout the study.

## Results

### Treatment interventions

Of the patients in the study, 94.3% received some sort of outpatient treatment and 20.7% received inpatient care. Individual therapy was the most common outpatient treatment, followed by family-based treatment and somatic treatment (see Table 1). Treatment interventions were also examined in three age groups; (1) 13–14 years, (2) 15–17 years and (3) 18–19 years. The percentage treated with individual therapy increased with every age group (57.8%, 75.6%, 84.6%), while the opposite could be said for family-based treatment (80.4%, 72.0%, 36.4%) and somatic treatment (59.0%, 53.6%, 41.8%). For individual therapy and family-based treatment the difference was significant for all three age groups, whereas for somatic treatment the difference was significant between groups 2–3 and 1–3. The least common treatment intervention was group therapy, with no significant differences between the age groups. Neither did the amount of inpatient care differ across the age groups.

**Table 1:** Type of treatment interventions received and amount of sessions/days in treatment, registered at 1-year follow-up (*n* = 1899)

	Patients *n* (%)	Sessions M (SD)
***Outpatient treatment***	1791 (94.3)	45.2 (62.9)
Individual therapy	1403 (73.9)	15.7 (18.7)
Family-based treatment	1227 (64.6)	11.2 (10.0)
Group therapy	153 (8.1)	7.3 (8.1)
***Complementary interventions***	1298 (68.4)	17.3 (33.0)
Diet counseling	531 (40.9)	
Meal training	259 (20.0)	
Physiotherapy	361 (27.8)	
Somatic treatment	982 (51.8)	
Other treatment	395 (20.8)	

		Days M (SD)
***Inpatient care***	393 (20.7)	55.5 (49.0)
***Medical treatment***	591 (31.1)	257.3 (184.8)

*M* mean, *SD* standard deviation

Table 2 shows a two-step cluster solution with four clusters. This solution yielded a ratio of sizes (largest cluster to smallest cluster) of 1.86. The silhouette coefficient was 0.6, which is considered good quality. Cluster sizes were (1) 25.1% (*n *= 471), (2) 20.4% (*n *= 383), (3) 19.1% (*n *= 357) and (4) 35.4% (*n *= 663). A total of 1.3% (*n *= 25) were excluded from the cluster solution due to missing values. The importance of the cluster predictors in decreasing order was: individual therapy 1.0, family based treatment 0.72, inpatient care 0.49, group therapy 0.45, health-care consumption (basic interventions) 0.19 and health-care consumption (complementary interventions) 0.14.

**Table 2:** Demographic and clinical data distributed on treatment clusters

	Total sample *n *= 1899	Family-based treatment and/or inpatient care (FTIC) *n *= 471/25.1%		Extensive mixed treatment (EMT) *n *= 383/20.4%		Individual treatment (IT) *n *= 357/19.1%		Family-based and individual treatment (FIT) *n *= 663/35.4%		Sign.
Treatment onset										
Women *n* (%)	1787 (94.1)	446 (94.7)		366 (95.6)		337 (94.4)		638 (96.2)		0.496
Age M (SD)	16.1 (1.8)	15.4 (1.8)		16.1 (1.8)		17.1 (1.6)		16.0 (1.6)		< 0.001
AN *n* (%)	1050 (55.3)	295 (62.6)		257 (67.1)		160 (44.8)		324 (48.9)		< 0.001
Normal weight *n* (%)	840 (44.2)	194 (41.2)		128 (33.4)		174 (48.7)		327 (49.3)		< 0.001

	*n* (%)	*n* (%)	% of total	*n* (%)	% of total	*n* (%)	% of total	*n* (%)	% of total	
Treatment										
Individual therapy	1403 (73.9)	0 (0)	0	369 (96.3)	19.4	357 (100)	18.8	663 (100)	35.0	< 0.001
Family based therapy	1227 (64.6)	283 (60.1)	14.9	265 (69.2)	14.0	0 (0)	0	663 (100)	35.0	< 0.001
Group therapy	154 (8.1)	0 (0)	0	152 (39.7)	8.0	0 (0)	0	0 (0)	0	< 0.001
Complementary interventions	1299 (68.4)	258 (54.8)	13.6	287 (74.9)	15.1	197 (55.2)	10.4	540 (81.4)	28.4	< 0.001
Inpatient treatment	393 (20.7)	156 (33.1)	8.2	237 (61.9)	12.5	0 (0)	0	0 (0)	0	< 0.001
Premature termination of treatment	184 (9.7)	49 (10.4)		25 (6.5)		62 (17.4)		46 (6.9)		< 0.001
Number of sessions M (SD)										
Health care consumption (basic)	45 (63)	29 (33)		86 (111)		29 (31)		40 (34)		< 0.001
Health care consumption (complementary)	12 (29)	6 (10)		29 (56)		5 (9)		10 (13)		< 0.001
Outcome at 1-year follow-up										
Remission *n* (%)	753 (39.7)	231 (49.0)		132 (34.5)		121 (33.9)		260 (39.2)		< 0.001
Normal weight *n* (%)	1205 (63.4)	308 (65.4)		228 (59.5)		239 (66.9)		415 (62.6)		0.138
Sick leave *n* (%)	57 (3.0)	13 (2.8)		24 (6.3)		6 (1.7)		13 (2.0)		< 0.001
Treatment completion *n* (%)	681 (36.3)	173 (36.7)		98 (25.6)		164 (45.9)		246 (37.1)		< 0.001

*M* mean, *SD* standard deviation

Cluster 1 was “family-based treatment and/or inpatient care” (FTIC), which consisted of the youngest individuals who received family-based treatment and/or inpatient care as a main treatment and had quite a low rate of health-care consumption. Cluster 2 was “extensive mixed treatment” (EMT), which had the highest rates of health-care consumption and was the only group who received a mix of all treatment forms, including group therapy and inpatient care. Cluster 3 was “individual therapy” (IT), which consisted of the oldest individuals who received individual therapy as a main treatment, of which more than half had subthreshold AN. Cluster 4 was “family-based and individual treatment” (FIT), which was the largest cluster and consisted of individuals who received a mix of those treatment forms, although not significantly distinguishable in any other aspect.

The proportion of patients with a normal weight at treatment onset was lowest in EMT (significantly lower than in IT and FIT), and the proportion of patients diagnosed with AN was the highest in EMT (together with FTIC, significantly higher than in IT and FIT). The proportion of patients who ended treatment prematurely was highest in IT (significantly higher than in EMT and FIT). There were no significant differences across clusters regarding gender distribution.

At 1-year follow-up, FTIC had the largest proportion of patients in remission (significantly larger than in EMT and IT), and the proportion of patients on sick leave was highest in EMT (significantly higher than in FIT). IT had the largest proportion of individuals who had completed treatment at 1-year follow-up (significantly higher than in EMT and FIT). There were no significant differences across clusters regarding weight status at 1-year follow-up.

The regression analysis showed that patients in EMT and FIT had a decreased chance of achieving remission at 1-year follow-up compared to patients in FTIC (see Table 3). Although not significant in the adjusted analysis, the results indicate that patients in IT also were less likely to achieve remission compared to patients in FTIC. The analysis also showed that patients who terminated treatment prematurely had a decreased chance of achieving remission.

**Table 3:** Logistic and multiple logistic regression analyses with remission as an outcome variable

	In remission (*n* = 753)	Not in remission (*n* = 1146)	Unadjusted		Adjusted	
			β (95% CI)	Sign.	β (95% CI)	Sign.
Age at treatment onset M (SD)	15.9 (1.7)	16.2 (1.8)	0.90 (0.86 0.95)	< 0.001	0.95 (0.89 1.00)	0.061
AN (onset) (%)						
Yes	401 (53.3)	649 (56.6)	Ref.	0.148	1.18 (0.53 1.49)	0.166
No	352 (46.7)	497 (43.4)	1.15 (0.95 1.38)			
Low weight (onset) (%)						
Yes	402 (53.4)	657 (57.3)	Ref.	0.091	1.06 (0.83 1.34)	0.665
No	351 (46.6)	489 (42.7)	1.17 (0.98 1.41)			
Cluster, based on treatment interventions and health care consumption (%)						
FTIC	231 (31.0)	240 (21.2)	Ref.			
EMT	132 (17.7)	251 (22.2)	0.55 (0.41 0.72)	< 0.001	0.54 (0.41 0.72)	*< 0.001*
IT	121 (16.3)	236 (20.9)	0.53 (0.40 0.71)	< 0.001	0.60 (0.44 0.81)	0.001
FIT	260 (34.9)	403 (35.7)	0.67 (0.53 0.85)	0.001	0.64 (0.50 0.82)	*< 0.001*
Premature termination of treatment (%)						
Yes	33 (4.4)	151 (13.2)	0.30 (0.21 0.45)	< 0.001	0.30 (0.20 0.41)	*< 0.001*
No	720 (95.6)	995 (86.8)	Ref.			

Italic values indicate significance of p value (p < 0.001)

*M* mean, *SD* standard deviation

### Patient satisfaction

The patients who received individual therapy were most satisfied (92.6% rated the treatment form as “very helpful” or “somewhat helpful”). The corresponding numbers for the remaining treatment forms were: group therapy (85.1%), family-based treatment (83.2%), inpatient care (81.4%) and complementary interventions (70.0%). When examining patient satisfaction in the different clusters we found that patients in EMT (*n *= 110) and FIT (*n *= 180) rated individual therapy higher than family-based treatment.

The majority of patients (77.4%) reported an overall improvement in issues with food, weight, and eating 1 year after entering treatment. The largest proportion of reported improvements could be seen in eating habits (84.6% in “impulses to avoid eating,” 77.6% in “anxiety before meals,” and 79.5% in “irregular and insufficient meals”). The proportion of reported improvements regarding compulsive physical activity was slightly smaller (66.9% in “impulses to be constantly active and in motion” and 58.6% in “excessive physical activity”). The proportion of reported improvements regarding thought processes were 68.6% in “consistent thoughts of food and weight,” 61.6% in “fear of gaining weight,” and 51.1% in “feeling fat and chubby”.

**Fig. 2:**
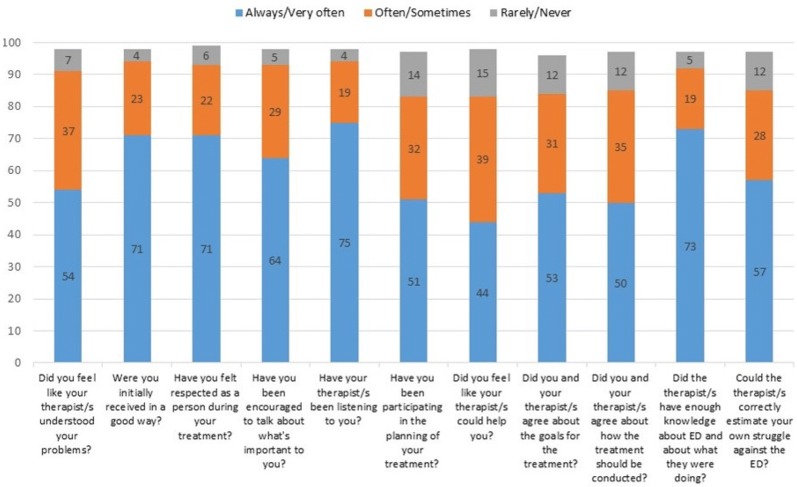
Patients’ evaluations of their therapist/s. Estimations in percent (%), total number of patients between 457 and 464 for every question

The patients evaluated the therapist’s ability to listen to them and their knowledge about ED highly, but gave lower ratings to the therapist’s ability to help them, their own participation in the planning of the treatment and their agreement with the therapist/s about how the treatment should be conducted (see Fig. 2).

**Fig. 3:**
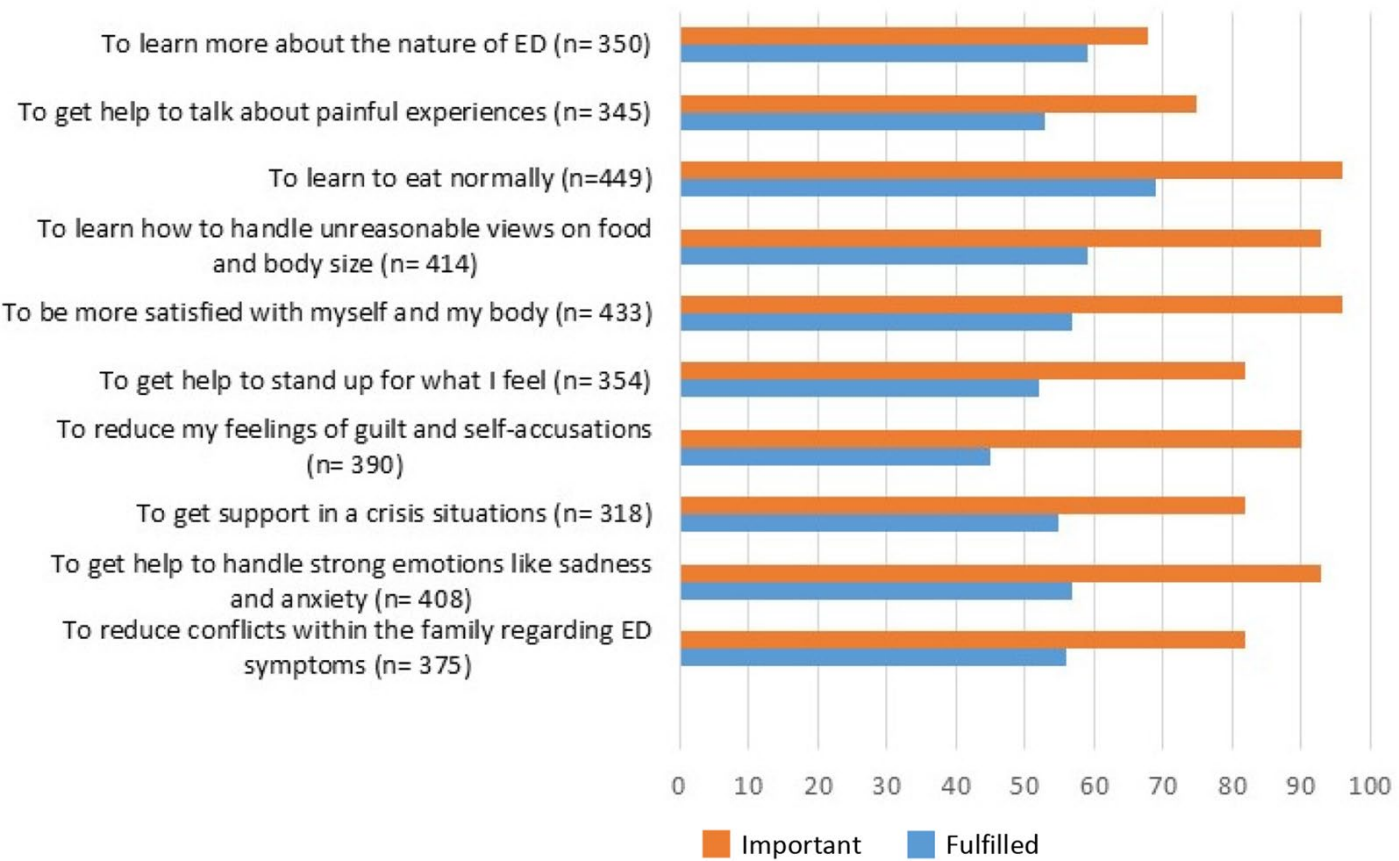
Importance and fulfillment of goals with treatment. How large a proportion of the patients felt the goal was very important or important and considered it completed or almost completed

The treatment goal that the patients evaluated as most important was “to learn to eat normally” (84% rated it as “very important” and 12% rated it as “important”). That was also a rather highly rated goal concerning fulfillment (70% rated it as “completed” or “almost completed”) (see Fig. 3). There were significant differences between the proportions of patients who felt a certain goal was important and the ones who considered the goal fulfilled, regarding all goals except “to learn more about the nature of ED.”

There were no significant differences across the clusters regarding improvements in overall ED symptoms, satisfaction with therapist relationship and perceived fulfillment of treatment goals. Significantly fewer patients among those who terminated treatment prematurely considered treatment interventions helpful (69.0% vs. 92.4%), and were less satisfied with fulfillment of treatment goals (26.9% vs. 63.9%). However, the results are uncertain due to the small proportion of participants who terminated treatment prematurely and responded to the patient satisfaction questionnaire (*n* = 30).

## Discussion

Individual therapy was the most common intervention in the total study population, followed by family-based treatment. This result is in contrast to previous studies which have suggested that family-based treatment is the most common intervention among adolescents with a restrictive ED [10, 15]. The incidence of individual therapy increased with the patients’ age and the incidence of family-based treatment decreased, and the fact that FIT (which included patients who received a combination of these treatment forms) was the largest cluster indicates that many patients received a combination of individual and family-based treatment sessions. The main focus in family-based treatment, in addition to restoring weight and normalizing food intake, is on interpersonal processes within the family [10, 15, 17], with adaptations to different age groups [50]. Thus, the need for family-based treatment might decrease in later adolescence, as the young people become more independent and less reliant on their family [51]. At the same time, the need for additional individual treatment sessions might increase, including a broader focus on adolescents’ social contexts outside the family [52].

Patients in the cluster FTIC, who received mainly family-based treatment and/or inpatient care and had the lowest age in average, were most likely to achieve remission at 1-year follow-up, compared to patients in the other clusters. This is in line with previous evidence for the benefits of identifying early symptoms and inserting treatment at an early stage [1, 53]. Furthermore, the result indicates that the combination of interventions in FTIC, with family-based treatment as one of the main treatments, is effective for young patients.

Severe ED, often in combination with psychiatric comorbidity, generally requires comprehensive treatment efforts and extended treatments [6, 54]. Patients in cluster EMT had the largest amount of health-care consumption and received a mix of all treatment forms, including inpatient care. Most of the patients were diagnosed with AN, and had a low weight at treatment onset. The remission rate at 1-year follow-up was low, and only a small proportion of patients had completed treatment. The proportion of patients on sick leave at 1-year follow-up was high. Most likely, patients in EMT needed more time to achieve remission than 1 year in treatment. In a previous study, examining treatment outcome and treatment duration in adolescents registered in SwEat, the average treatment duration was 15 months. The results indicated that longer treatment duration was positively correlated to higher remission rates [33].

Cluster IT, with the highest mean age and a large proportion of patients who were diagnosed with subthreshold AN, had an even lower remission rate at 1 year follow-up. However, almost half of the patients in cluster IT had completed treatment by the time for 1-year follow-up. This might illustrate difficulties in finding the right treatment for patients with a more diffuse ED symptomatology [55], or practical difficulties for older adolescents in combining treatment with other areas in life, such as school and spare-time activities [52].

Not surprisingly, the results showed that whether treatment was discontinued or followed according to plan predicted outcome at 1-year follow-up in terms of being in remission or not. Patients who terminated treatment prematurely had a decreased chance of achieving remission. Premature termination of treatment might be associated with treatment dissatisfaction, which in turn might lead to slow treatment progress and an increased risk of relapse [25, 31, 32]. This is supported by results from the present study, indicating that patients who terminated treatment prematurely were significantly more dissatisfied with their treatment and with fulfillment of treatment goals. This points to the importance of learning more about how patient satisfaction can be promoted [31].

Among those who answered the patient satisfaction questionnaire, the patients who received individual therapy were generally more satisfied with their treatment. The patients in the clusters EMT and FIT, which included both individual therapy and family-based treatment, rated individual therapy higher than family-based treatment, and patients in EMT, IT and FIT rated individual therapy higher than complementary interventions. Patients’ perceived importance of individual treatment sessions has been pointed out in earlier studies [23, 24, 26], and patients have been arguing for the possibility of getting at least one individual treatment session as part of their treatment [26]. In the present study, the fact that family-based treatment was less appreciated by the adolescents might have to do with the nature of such treatment form, in which the young peoples’ own thoughts and opinions are given less priority, in favor of joint conversations with the family. In addition, family-based treatment would possibly gain from adapting better to a society in change. Such adaption might include bringing patients’ other social contexts into treatment to a larger extent, and adjusting treatment to different family constellations [52].

Most of the patients who answered the patient satisfaction questionnaire perceived the form of treatment they received as helpful. The most important treatment goal, from the patients point of view was “to learn to eat normally”, which was also rated high concerning fulfillment. This goes well with the actual groundwork in ED treatment, which includes meal planning and striving for a regular and sufficient eating [10]. However, while most patients reported improvements in behavioral symptoms, far fewer patients felt that they had markedly improved in relation to thoughts of food and weight, fear of gaining weight, and body perception. Rigid thoughts are parts of the core symptomatology [44], and often lead to resistance in treatment [56]. Working with rigid thoughts and underlying psychological factors in treatment has proven to be particularly important in this diagnose group, in order to achieve a good treatment outcome and decrease the risk of relapse [26, 29, 30, 57]. There seem to be room for improvement in that regard since therapists’ ability to help was rated quite low by the patients.

Even if the majority of the responding patients in general were quite satisfied with the therapist relationship, they rated the following aspects low: the therapist’s ability to help them, their own participation in the planning of the treatment, the therapist’s ability to understand their problems and estimate their own struggle against the ED, and their agreement with the therapist/s about treatment goals and how the treatment should be conducted. This is in line with results from previous studies, suggesting that therapist alliance in adolescent treatment is difficult to achieve [58, 59]. Nevertheless, adolescents in general place great emphasis on the therapist relationship [30, 33], and therapist alliance has proven to be crucial for a good treatment outcome [60, 61]. The ego-syntonic aspect of AN, and patients’ denial of illness sometimes poses a real challenge to therapists [59]. In such cases, the families’ engagement in treatment might constitute an important substitute. In family-based treatment, the parents’ alliance with the therapist often becomes stronger than that of the patients’ [24, 59].

Patients’ wish to be listened to in treatment has been highlighted previously, and a sound therapeutic dialogue seems to be a prerequisite for a well-established therapeutic alliance [59, 62]. Patients’ low rating of their therapist’s ability to help them indicates a lack of trust, but might have to do more with the patients’ low confidence in their own ability to recover. In general, therapists need to involve patients in the planning of their treatment to a larger extent, which implies talking more openly, asking the patients about their opinions and expectations [63, 64], and, when possible, letting the patients have more to say about the treatment plan [26, 65, 66].

Goals that more than 90% of the patients rated as important were “to learn how to handle unreasonable views on food and body size,” “to be more satisfied with myself and my body,” and “get help to handle strong emotions like sadness and anxiety,” but fewer than 60% of the patients considered these goals completed or almost completed. This points to the importance of updated treatment interventions focusing on body image, self-perception and emotion regulation. In a previous qualitative study, patients themselves argue for the benefits of such focus in treatment [67].

### Strengths and limitations

A strength of this study is its naturalistic design, lending the results high external validity and allowing a generalizability to a clinical environment [68]. Another strength is the number of participating units, which provides good national coverage. Nevertheless, the naturalistic design also implies major limitations [9, 69]. One example is lack of control over the assessments of symptoms and diagnoses and the content of different treatment forms at different units. The fact that we do not have any reliable data on psychiatric comorbidities is an obvious limitation, although we have no reason to believe that comorbidity would be different in the different study samples or clusters.

Considerable attrition at follow-up in SwEat, and low response rates regarding the patient satisfaction questionnaire are limitations over which we had no control when designing the study. In addition, the two study populations differ remarkably in size, despite coming from the same initial sample. Participating in the 1-year follow-up, as well as filling in the patient satisfaction questionnaire, is voluntary for the patients, but the follow-up registration is part of the patient administration and conducted by patients and the therapists together, whereas the patient satisfaction questionnaire is sent home to the patients to fill in by themselves. In other words, more responsibility is put on the patients in the patient satisfaction registration, which might explain the low response rate and the small sample size. Another possible explanation for the large amount of missing data is that forms that are filled in manually, which was the case for the patient satisfaction questionnaires during the first years examined, are sometimes difficult to interpret.

The low response rate regarding the patient satisfaction questionnaire decreases the generalizability of the results, and there is a risk that the results are misrepresentative to some extent. Patients who have stronger opinions about their treatment, both positive and negative, might be more likely to answer a patient satisfaction questionnaire than patients who have more neutral opinions. It has been shown that high participation rates in similar surveys generally correlate to a good outcome, although the effects of non-response bias on final results often are quite small [70]. Some questions in the questionnaire have a larger number of missing or invalid answers than others, which might be due to that patients tend to skip questions that do not apply to them.

An obvious limitation is the fact that the patient satisfaction questionnaire has not been validated, although it can be considered to have face validity as it was developed by clinicians who are well experienced in working with ED patients.

Despite the missing registrations, the large sample sizes in the present study can be considered a strength. In total, we have valuable information about approximately 2000 individuals, providing a good representation of the total population, and responses from nearly 500 young patients, which may indicate possible response patterns in a larger population.

## Conclusions

Young adolescents who received mainly family-based treatment and/or inpatient care seemed to respond more rapidly to treatment compared to older adolescents who more often received individual therapy or mixed treatment interventions.

Individual therapy was the most common treatment form, followed by family-based treatment and somatic treatment, and among the patients who answered the patient satisfaction questionnaire, individual therapy was the most appreciated treatment form.

In general, the adolescent patients were quite satisfied with their therapist relationships, but they gave low ratings to statements associated with their own participation in treatment.

Although most patients of those who answered the patient satisfaction questionnaire reported improvements in their attitude to food and eating, and their ability to eat regularly and sufficiently, far fewer patients felt that they had improved regarding consistent thoughts of food and weight, and fear of gaining weight, which are symptoms of great importance for long term recovery.

The results from the present study contribute to the existing knowledge about treatment interventions, treatment outcome and patient satisfaction in adolescents with AN or subthreshold AN. However, further research is needed to examine patients over longer follow-up intervals using different research methods, with an aim to learn more about the value of different treatment forms and patient satisfaction for clinical improvements.

### Clinical implications

- Young adolescents who receive mainly family-based treatment and/or inpatient care seem to respond more rapidly to treatment compared to older adolescents who receive individual therapy or mixed treatment interventions.
- It is important to focus on cognitive symptoms in treatment. Although most patients of those who answered the patient satisfaction questionnaire reported improvements in their attitude to food and eating, and their ability to eat regularly and sufficiently, far fewer patients felt that they had improved regarding consistent thoughts of food and weight, and fear of gaining weight, which are symptoms of great importance for long-term recovery.
- Patients should be involved in the planning of their treatment, or at least receive information on the decisions taken. In the present study, the adolescent patients were quite satisfied with their therapist relationships, but they gave low ratings to statements associated with their own participation in treatment.

## Supplementary information

**Additional file 1.** Register variables.

## Data Availability

Data from the current study are not publicly available in order to maintain participant confidentiality, but are available from the corresponding author on reasonable request with approval from the relevant institutional review board.

## Abbreviations

AN: Anorexia nervosa
ED: Eating disorders
SwEat: Swedish national quality register for eating disorder treatment
EOT: End of treatment
DSM-IV: Diagnostic and Statistical Manual of Mental Disorders IV
SEDI: Structured Eating Disorder Interview
RAB: Rating of Anorexia and Bulimia Interview
EDNOS: Eating disorder not otherwise specified
DSM-5: Diagnostic and Statistical Manual of Mental Disorders 5
BIC: Schwarz’s Bayesian Criterion
ANOVA: Analysis of variance
FTIC: Family-based treatment and/or inpatient care
EMT: Extensive mixed treatment
IT: Individual therapy
FIT: Family-based and individual treatment
