# Supplementary material for: Adolescents with full or subthreshold anorexia nervosa in a naturalistic sample: treatment interventions and patient satisfaction
Source: Child Adolesc Psychiatry Ment Health. 2020 May 2;14:16. doi: 10.1186/s13034-020-00323-9 (PMC7196214; doi:10.1186/s13034-020-00323-9)
Supplement: Supplementary file 1 — Additional file 1. Register variables. [file 13034_2020_323_MOESM1_ESM.docx]

**Additional file 1**

Data collected from SwEat at one-year follow-up registration^1^

| **Follow-up registration** |
| --- |
| Gender |
| Age at first ED symptoms (years) |
| Treatment interventions during the last year – outpatient treatment (Individual treatment/Family treatment/Group therapy/Complementary interventions)/No outpatient treatment – Number of sessions |
| Treatment interventions during the last year – inpatient care (Specialized ED/General psychiatric/General somatic)/No inpatient care – Number of days |
| Treatment interventions during the last year – medical treatment (Sedatives-Hypnotics/Antidepressants/ Neuroleptic/ Other)/No psychopharmaceutic treatment) - Number of days |
| The patient’s current weight (kg, to one decimal) |
| The patient’s current height (cm, to one decimal) |

^1^This table only includes data presented in the study. The original SwEat one-year follow-up registration contains additional data.

Data collected from SwEat patient satisfaction registration^1^

| **Patient satisfaction questionnaire** |
| --- |
| State below which form of treatment intervention you have received during the last year and estimate, where applicable, how valuable the treatment has been:  (Answer alternatives: Yes/No/Very helpful, Somewhat helpful, Not helpful and The problem got worse) |
| - Regular talks between you and a therapist - Talks with your family together with a therapist - Group discussions together with other persons with ED - Diet consultation - Eating/Meal training - Physiotherapy - Doctor consultation about your bodily health - Day care - Inpatient care at a specialized ED department - Inpatient care at a general psychiatric department - Inpatient treatment at a general somatic department - Medication (e.g. sedatives and antidepressants) - Other treatment |
| Questions about the contact between you and your therapist/s during the last year:  (Answer alternatives: Always, Very often, Often, Sometimes, Rarely and Never)  (In the analysis, the answers were trichotomized into: Always/Very often, Often/Sometimes and Rarely/Never – see Figure 2.) |
| - Did you feel like your therapist/s understood your problems? - Were you initially received in a good way? - Have you felt respected as a person during your treatment? - Have you been encouraged to talk about what´s important to you? - Have your therapist/s been listening to you? - Have you been participating in the planning of your treatment? - Did you feel like your therapist/s could help you? - Did you and your therapist/s agree about the goals for the treatment? - Did you and your therapist/s agree about how the treatment should be conducted? - Did the therapist/s have enough knowledge about ED and about what they were doing? - Could the therapist/s correctly estimate your own struggle against the ED? |
| Below follows a number of goals for treatment of eating disorders that could have been more or less important to you. State if a goal has been relevant to you and, where applicable, estimate how important the goal has been to you and to what degree it has been fulfilled:  (Answer alternatives: Relevance: Yes/No Importance; Very important/Important/Not so Important and Not Important Fulfillment of goal: Yes, completely/Yes, almost/To some extent and Not at all)  (In the analysis, the answers were dichotomized into: Very important/Important and Completely fulfilled/almost fulfilled – see Figure 3.) |
| - To learn more about the nature of eating disorders - To get help to talk about painful experiences - To learn to eat normally - To learn how to handle unreasonable views on food and body size - To be more satisfied with myself and my body - To get help to stand up for what I feel - To reduce my feelings of guilt and self-accusations - To get support in crisis situations - To get help to handle strong emotions, like sadness and anxiety - To reduce conflicts within the family regarding ED symptoms |
| Compared with how it was one year ago; how is it now considering:  (Answer alternatives: Much worse, Worse, Somewhat worse, Unchanged, Somewhat better, Better, Much better, Not relevant)  (In the analysis, the answer alternatives were trichotomized into: Not improved/Unchanged/Improved, and the statements were divided into the categories eating habits, excessive physical activity and thought processes.) |
| - Anxiety before meals - Impulses to avoid eating - Consistent thoughts of food and weight - Irregular and insufficient meals - Fear of gaining weight - Feeling fat and chubby - Impulses to be constantly active and in motion - Excessive physical activity - Overall issues with food, eating and weight |

^1^ This table only includes data presented in the study. The SwEat patient satisfaction questionnaire contains additional data.
